# Genetic Alterations and Transcriptional Expression of m^6^A RNA Methylation Regulators Drive a Malignant Phenotype and Have Clinical Prognostic Impact in Hepatocellular Carcinoma

**DOI:** 10.3389/fonc.2020.00900

**Published:** 2020-07-21

**Authors:** Gui-Qi Zhu, Lei Yu, Yu-Jie Zhou, Jun-Xian Du, Shuang-Shuang Dong, Yi-Ming Wu, Ying-Hong Shi, Jian Zhou, Jia Fan, Zhi Dai

**Affiliations:** ^1^State Key Laboratory of Genetic Engineering, Liver Cancer Institute, Zhongshan Hospital, Fudan University, Shanghai, China; ^2^Key Laboratory of Carcinogenesis and Cancer Invasion, Fudan University, Ministry of Education, Shanghai, China; ^3^Department of Liver Surgery and Transplantation, Liver Cancer Institute, Zhongshan Hospital, Fudan University, Shanghai, China; ^4^Key Laboratory of Gastroenterology and Hepatology, Ministry of Health, Shanghai, China; ^5^Division of Gastroenterology and Hepatology, Renji Hospital, School of Medicine, Shanghai Institute of Digestive Disease, Shanghai Jiao Tong University, Shanghai, China; ^6^Department of General Surgery, Zhongshan Hospital, Fudan University, Shanghai, China; ^7^Department of Urology, The Second Affiliated Hospital and Yuying Children's Hospital of Wenzhou Medical University, Wenzhou, China

**Keywords:** RNA modification, m^6^A, hepatocellular carcinoma, TP53 mutation, prognosis

## Abstract

**Background:** N6-methyladenosine (m^6^A) RNA methylation, associated with cancer initiation and progression, is dynamically regulated by the m^6^A RNA regulators. However, its role in liver carcinogenesis is poorly understood.

**Methods:** Three hundred seventy-one hepatocellular carcinoma (HCC) patients from The Cancer Genome Atlas database with sequencing and copy number variations/mutations data were included. Survival analysis was performed using Cox regression model. We performed gene set enrichment analysis to explore the functions associated with different HCC groups. Finally, we used a machine-learning model on selected regulators for developing a risk signature (m^6^Ascore) The prognostic value of m^6^Ascore was finally validated in another two GEO datasets.

**Results:** We demonstrated that 11 m^6^A RNA regulators are significantly differentially expressed among 371 HCC patients stratified by clinicopathological features (P<0.001). We then identified two distinct HCC clusters by applying consensus clustering to m^6^A RNA regulators. Compared with the cluster2 subgroup, the cluster1 subgroup correlates with poorer prognosis (*P* < 0.001). Moreover, the cell cycle, splicesome and notch signaling pathway are significantly enriched in the cluster1 subgroup. We further derived m^6^Ascore, using four m^6^A regulators, predicting HCC prognosis well at three (AUC = 0.7) or 5 years (AUC=0.7) in validation. The prognostic value of m^6^Ascore also was validated successfully in two GEO datasets (*P* < 0.05). Finally, we discovered that mutations and copy number variations of m^6^A regulators, conferring worse survival, are strongly associated with TP53 mutations in HCC.

**Conclusions:** We find a significant relationship between the alterations and different expressions causing increased m^6^A level and worse survival, especially in TP53-mutated HCC patients. Genetic alterations of m^6^A genes might cooperate with TP53 and its regulator targets in the HCC pathogenesis. Our m^6^Ascore may be applied in the clinical trials for patient stratification in HCC.

## Introduction

Hepatocellular carcinoma (HCC) is the fifth leading cause of malignant cancer and the third most common reason for cancer-specific death worldwide (1, 2). The HCC mortality is often high because of metastasis and postsurgical liver recurrence. Hence, effective treatments are eagerly awaited to hold back additional metastases to improve the disappointing HCC outcomes (2). However, advanced HCC with recurrence or low response to chemotherapy have low overall survival nowadays. The lack of effective interventions and high mortality of HCC demand a better understanding of the cancer molecular mechanism.

N6-methyladenosine (m^6^A), first described in 1974, the most abundant form of internal messenger RNA (mRNA) modification in higher eukaryotes, has become of great interest in recent years (3). It is known to play vital part in regulating gene expression, splicing in cellular biology, and cellular protein translation (3–5). The m^6^A regulators comprise “writers” such as methyltransferase like 3 (METTL3), WT1-associated protein (WTAP), METTL14, RNA binding motif protein 15 (RBM15), zinc finger CCCH domain-containing protein 13 (ZC3H13) and KIAA1429 (also known as VIRMA). “Readers” such as YTH domain-containing 1 (YTHDC1), YTH N6-methyladenosine RNA binding protein 2 (YTHDF2), YTH domain-containing 1 (YTHDC2), heterogeneous nuclear ribonucleoprotein C (HNRNPC), YTH N6-methyl-adenosine RNA binding protein 1 (YTHDF1) and ‘erasers' such as fat mass- and obesity-associated protein (FTO) and α-ketoglutarate-dependent dioxygenase alkB homolog 5 (ALKBH5) (6–12). m^6^A dysregulation regulated by knockdown of genes could result in decreased cell proliferation, cell death and developmental defects (3).

In recent years, increasing amounts of evidence showed that genetic changes and dysregulated expression of m^6^A RNA methylation genes are correlated with malignant phenotype closely in different types of cancer (7, 13–17). For example, knockout of METTL3 disturbs embryonic stem cell differentiation (18). Depletion of erasers, such as FTO and ALKBH5, can result in obesity and dysregulation of spermatogenesis (3, 9). Knockdown of m^6^A methyltransferase can cause regulation of the TP53 signaling pathway relevant to tumorigenesis (4). Recently, the overexpression of METTL3 result in HCC tumor progression by repressing SOCS2 through the m^6^A-YTHDF2 axis in HCC (19). Additionally, down-regulation of METTL14 as a dismal prognostic factor for HCC overall survival (20). Therefore, it is surprising that the profile of genetic alterations affecting m^6^A regulatory genes and gene expression of corresponding m^6^A genes have not been explored in HCC.

Hence, in our study, we systematically evaluated the genetic alterations and expression of 13 widely reported m^6^A RNA regulators with RNA sequencing data from The Cancer Genome Atlas (TCGA) (*n* = 377) datasets. We analyzed the alteration spectrum and expression of every m^6^A modification regulator with regards to different clinicopathological factors, including survival.

## Materials and Methods

### Patient Data

The datasets GSE14520 and GSE63898 were downloaded from the expression database GEO (Gene Expression Omnibus, http://www.ncbi.nlm.nih.gov/geo/) (21, 22). GSE14520 included a total of 488 samples, 241 samples were paired non-tumor samples, while the other 247 samples were HCC samples. Platform Information was [HG-U133A_2] Affymetrix Human Genome U133A 2.0 Array for 43 samples, and [HT_HG-U133A] Affymetrix HT Human Genome U133A Array for the other 445 samples. GSE63898 included 228 HCC and 168 cirrhotic samples, and the platform was [HG-U219] Affymetrix Human Genome U219 Array for all samples. The clinicopathological, mutation, deletion, amplification, copy number variation and/or survival data from HCC patients are available via the cBioportal (23), the TCGA data portal and/or reported in a previous publication (24). Of the 424 HCC patients in the TCGA cohort, matched mutation, deletion, amplification and copy number variation (CNV) data are available for 366 patients via cBioportal (23). We therefore included only these patients in our genetic alteration analyses. In addition, of the 424 HCC patients included in our gene expression analysis, corresponding complete clinical information are available for 236 patients from the TCGA cohort.

### Selection of m^6^A RNA Methylation Regulators

We first collected 16 m^6^A RNA methylation regulators from published literature (7, 12, 16), and we retrieved the m^6^A genes with available gene expression from the HCC TCGA cohort. This brought a list of 13 m^6^A regulators. Finally, the genetic alterations and expression of m^6^A RNA methylation regulators in HCC with different clinicopathological factors were compared systematically.

### Statistical Analysis

For gene expression analysis, to investigate the function of m^6^A RNA methylation gene regulators in HCC, we clustered the HCC into different groups with “ConsensusClusterPlus” (resample rate of 80%, 50 iterations and Pearson correlation, http://www.bioconductor.org/). We used PCA with the R package for R v3.5.1 to explore the gene expression profiles among various HCC patient subgroups. We performed GO and KEGG pathway analyses with the Database for Annotation, Visualization, and Integrated Discovery to annotate differentially expressed genes in various HCC subgroups. We also analyzed interactions among m^6^A RNA methylation regulators from the STRING database (http://www.string-db.org/). We finally applied gene set enrichment analysis (GSEA) to evaluate the functions associated with different HCC subgroups. To determine the prognostic value of m^6^A RNA methylation genes, we therefore performed several univariate Cox regression analyses of their gene expression in the TCGA cohort. To this end, we confirmed nine genes that were significantly associated with patient survival (*P* < 0.05), which we then selected for further functional analysis and development of a risk signature (m^6^Ascore) with the machine learning algorithm model (25). Finally, four m^6^A regulatory genes and their coefficients were determined by R package “Coxboost” within the training set (All patients were randomly divided 75% for training and 25% for validation) by the machine learning model (25). The m^6^Ascore for the signature was calculated using the formula:

$$\begin{array}{l} {\text{m}}^{6}\text{Ascore}=\sum_{\text{i}=1}^{\text{n}}\text{Coe}{\text{f}}_{\text{i}}*{\text{x}}_{\text{i}}, \end{array}$$

where Coef_i_ is the coefficient, and x_i_ is the relative z-score-transformed gene expression of selected genes. We used the above formula to calculate a risk score for each HCC patient in the training (75% of TCGA patients) and internal validation (25% of TCGA patients) datasets.

Additionally, the loss and gain levels of CNVs have been confirmed using segmentation analysis and Genomic Identification of Significant Targets in Cancer algorithm (GISTIC) for CNV. To explore the clinical significance of the CNV or mutation, this TCGA cohort was divided into two HCC groups: “with mutation and/or CNV of 10 m^6^A regulatory” and “without CNV and mutation.” We calculated the gene expression from RNA-Seq V2 RSEM release, and used log scale before analyzing the associations between gene expression and CNVs.

Categorical variables applying the chi-square test or Fisher's exact test were compared. A Fisher-Freeman-Halton test was performed for contingency tables that are larger than 2 × 2. We used the Mann-Whitney *U*-test for comparing continuous variables that were not distributed normally. The Kaplan-Meier analysis and the log-rank test were used to estimate the distribution of overall (OS) and disease-free survival (DFS) and to compare differences between survival curves, respectively. We then performed multivariate analyses by applying the Cox proportional hazards regression models. Variables with *P* < 0.05 (log-rank test) in univariate analysis for OS were included in the further models. All statistical analyses were conducted by using the SPSS statistical package, version 24.0 (IBM, Corp.). In addition, because some comparisons were made using limited data, the statistical comparisons were correlated for multiple testing by R software. For all tests, statistical differences were considered as significant at *P* < 0.05 (two-sided).

## Results

### Genetic Alterations of m^6^A Regulators Predict Poorer Survival in HCC

**Figure 1:**
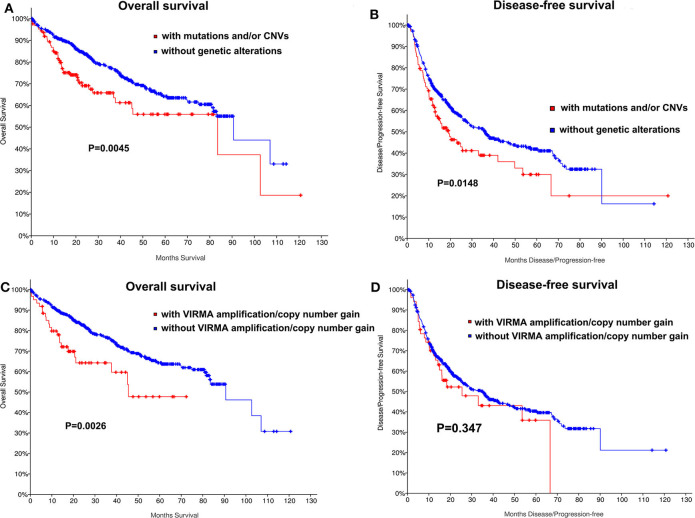
Genetic alterations of m^6^A regulatory genes in HCC. **(A)** Percentage of HCC samples with mutations or CNVs of the m^6^A regulators based on the data from TCGA. **(B)** Events of copy number gain or loss of m^6^A regulatory genes in HCC samples. **(C)** Relationships between Putative copy-number alterations and mRNA for FTO. **(D)** Relationships between Putative copy-number alterations and mRNA for ALKBH5.

Mutations of m^6^A regulatory genes were found in 8.5% (31/366) of HCC (Figure 1A). We identified gene variation in copy number in 23.0% (84/366) of patients (Table S1, detailed information in Table S2). A comparable frequency of copy number loss which is measured as shallow deletion by using GISTIC (*n* = 33) and copy number gain (*n* = 46) of m^6^A genes (Figure 1B). Copy number loss of VIRMA is the most frequent among the HCC cohort (59/366, 16%; Figure S1). Notably, 6.3% (23/366) of HCC patients obtained copy number loss and gain of more than one m^6^A genes simultaneously (Table S3). In seven of those 23 cases, an amplification of an m^6^A writer or m^6^A reader was investigated with a shallow or deep deletion of m^6^A eraser genes concomitantly (Table S3), which indicated a potential synergistic change of m^6^A enzymes that may result in increased levels of m^6^A RNA modification. Shallow deletions of ALKBH5 and FTO conferred reduced mRNA expression of these m^6^A genes significantly (Figures 1C,D). While copy number gain of METTL3, VIRMA, YTHDF1 and YTHDF2 conferred a significant increase in its RNA expression (Figure S2). Hence, copy number gain and shallow deletion might lead to the decreased and increased mRNA expression of m^6^A genes.

### Genetic Changes of m^6^A Genes Were Correlated to Clinicopathological and Molecular Characteristics

It was determined whether mutations or copy number variations (CNVs) of m^6^A regulators are correlated with HCC clinical and molecular characteristics. Mutations or CNVs of METTL14, METTL3, VIRMA, RBM15, ZC3H13, WTAP, YTHDC1, YTHDC2, YTHDF1, YTHDF2, HNRNPC, FTO, and ALKBH5 as a subgroup were significantly correlated with lower albumin (*P* = 0.038, Table S1), and poorer tumor stage in HCC (*P* < 0.0043, Table S1). In addition, we observed a significant increase in the status of TP53 mutations (*P* < 0.012, Table S1), TERT mutations (*P* = 0.018, Table S1), and ARID1A mutations (*P* = 0.047, Table S1) in HCC patients obtaining genetic changes of m^6^A genes. These above molecular characteristics also were correlate with mutations of m^6^A regulators alone, except for TERT mutations (Table S1). However, TERT (*P* = 0.011) and ARID1A mutations (0.0037), except for TP53 mutations (26) were also correlate with CNVs of m^6^A regulators alone (Table S1), which might be because of the small sample sizes for mutations and CNVs associated with most mutated HCC genes.

**Figure 2:**
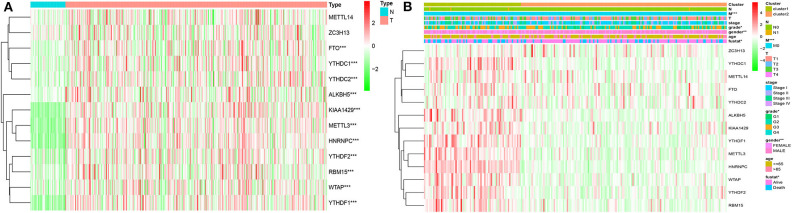
Kaplan-Meier curves for overall and disease-free survival of TCGA HCC patients by the presence and absence of **(A,B)** mutation and/or CNVs of m^6^A regulatory genes, **(C,D)** amplification/copy number gain of VIRMA.

We also determined whether shallow or deep deletion of VIRMA is correlate with the clinical and molecular characteristics. Consistent with our results for all m^6^A regulators, amplification/copy number gain of VIRMA was significantly correlated with poorer clinical stage and the presence of TP53 and TERT mutation in this HCC cohort (*P* =0.04, *P* = 0.027, respectively; Table S4). TP53 and TERT mutations were also present in HCC patients with amplification/copy number gain of VIRMA (Table S4). Kaplan-Meier analyses evaluating the impact of genetic changes for m^6^A regulators on OS and DFS in HCC patients were performed. As a group, HCC patients with the mutation of any of m^6^A regulators had a worse OS (*P* = 0.004) and DFS (*P* = 0.0148, Figures 2A,B). In addition, unfavorable OS were prominent in HCC patients who had amplification/copy number gain of VIRMA (Figures 2C,D).

Of all clinical and molecular characteristics regarded as the *de novo* HCC cohort, higher grade T classification (*T*>0), higher grade of stage and ARID1A, TP53 mutations and genetic alterations of any m^6^A genes were associated with poorer OS in univariate Cox analysis significantly (Table S5). Therefore, we investigated the impact of m^6^A gene mutations or CNVs on the HCC outcome with poor clinical characteristics. Changes of m^6^A genes as a subgroup were correlated with poorer OS in HCC patients regardless of stage, TP53, and ARID1A mutations (Table S6). These genetic alterations did not confer a worse OS in patients with higher grade T classification (*T*>0), tumor grade or ARID2, TERT mutations (Table S6). We then identified the HCC patients' survival according to whether they showed combined TP53 mutations and genetic changes of m^6^A genes. Almost half of the patients with mutated TP53 (40%, Table S1) had ≥1 genetic change of m^6^A gene. We further explored the gene expression of m^6^A regulatory genes between wild-type and mutated TP53. Interestingly, we found the m^6^A eraser genes showed significantly lower expression in mutated TP53 group than the wild-type group, but the m^6^A writer and reader genes showed significant higher expression in TP53 mutated group. It indicates that higher m^6^A levels among HCC patients were parallel with the mutation rates of TP53 during the carcinogenesis or initiation in HCC. The group of TP53 mutated patients had poorer OS than those patients not obtaining any of these genetic changes (Table S7).

### A Strong Correlation Between Genetic Alterations of m^6^A Genes and TP53 Mutations

Since mutations, amplifications, deletions, and/or CNVs of m^6^A genes were relatively restricted to patients with wild-type TERT and ARID1A (88.5%, Table S1), we then identified whether these genetic changes affect OS stratified by the status of TERT or ARID1A mutation. Poorer OS were seen in HCC patients with wild-type TERT who had more than one genetic change of m^6^A genes (*P* = 0.001, Table S7). Notably, these patients also have worse OS (*P* < 0.05) compared to those patients who had mutated TERT but had no genetic changes of m^6^A genes (Table S7). Genetic changes of m^6^A genes as a subgroup were also significantly associated with a worse OS in wild-type ARID1A patients (*P* = 0.009, Table S7). A combination of molecular analysis of m^6^A genes might be valuable to identify a worse outcome in HCC patients who had neither been classified as “poor risk” because of the presence of mutated TERT (27), nor better survival outcome conferred by ARID1A mutations (28, 29), particularly in the TP53 wild-type HCC patients. From a multivariate Cox analysis including variables correlated with worse survival, genetic changes of m^6^A genes, as a subgroup was not an independent factor for OS (Table S8). Genetic changes of m^6^A genes, however, predicted poorer OS independently (HR = 1.8; 95% CI, 1.2–4.4; *P* = 0.010) when the variable TP53 mutation was excluded from the original model (Table S8).

### Consensus Clustering of m^6^A RNA Methylation Regulators Identified Two Clusters of HCC With Distinct Clinical Outcomes and Characteristics

**Figure 3:**
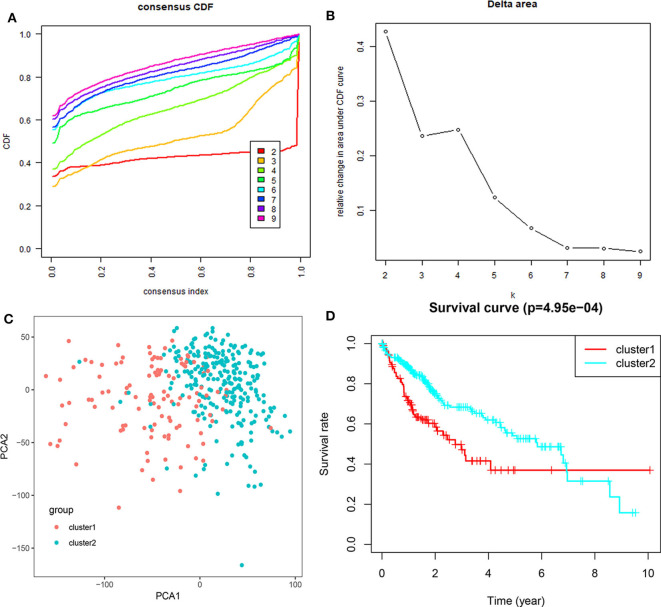
Expression of m^6^A RNA methylation regulators with different clinicopathological features in HCC. **(A)** stratified by tumor and normal samples; **(B)** stratified by cluster1/2 groups. *P < 0.01; **P < 0.001; ***P < 0.0001.

Considering the important biological functions of each m^6^A RNA genes in the HCC tumorigenesis, we explored the mRNA expression of each m^6^A regulators between tumor and normal tissues systematically. The expression level of each m^6^A RNA methylation regulator between tumor and normal are presented as heatmaps (Figures 3A,B), showing that the expressions of most m^6^A RNA methylation regulators are significantly higher in tumor than normal group (Figure 3 and Figure S3), except for METTL14 and ZC3H13 (but the expression still trends higher among tumor group than normal). We then validated these regulatory genes in our 69-paired HCC tumor and normal tissues by RT-PCR, which showed the same results as in the TCGA dataset (data were not shown).

**Figure 4:**
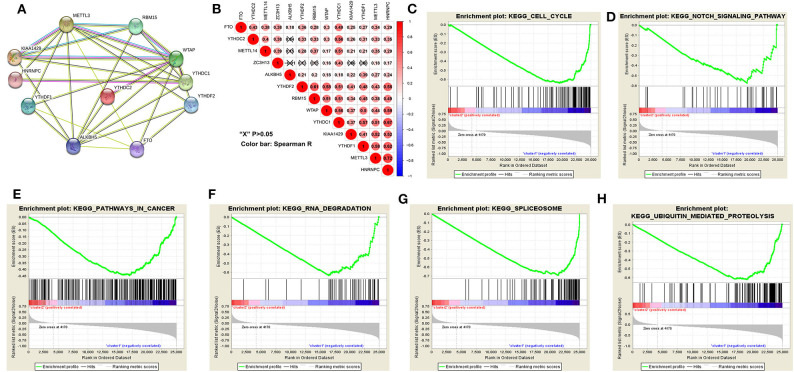
Differential clinicopathological features and overall survival of HCC in the cluster1/2 subgroups. **(A)** Consensus clustering cumulative distribution function (CDF) for *k* = 2–10. **(B)** Relative change in area under CDF curve for *k* = 2–10. **(C)** Principal component analysis of the total RNA expression profile in the TGGA dataset. **(D)** Kaplan–Meier overall survival (OS) curves for HCC patients according to cluster1/2.

Based on the expression similarity of m^6^A RNA regulators, *k* =3 seemed to be a suitable selection with clustering stability increasing from *k* = 2 to 10 in the TCGA datasets (Figure 4; Figure S4). However, we found that 116 out of 371 HCC patients clustered into one of these two groups in the TCGA dataset (Figure 3B). Hence, we compared the clinical characteristics of these two groups clustered by *k* = 2, including cluster1 and cluster2 (Figure 4D). The cluster2 subgroup is significantly correlated with no metastasis (*P* < 0.0001), lower grade (*P* < 0.01), and lower stage (*P* < 0.0001; Figure 3B). The cluster1 subgroup mainly contains HCC with higher grade and clinical stage at diagnosis. In addition, we observed a shorter OS in the cluster1 subgroup than the cluster2 group (median survival: 76.1 months vs. 90.1 months) (Figure 4D).

### Categories Identified by Consensus Clustering Are Closely Correlate to the Malignancy of HCC

**Figure 5:**
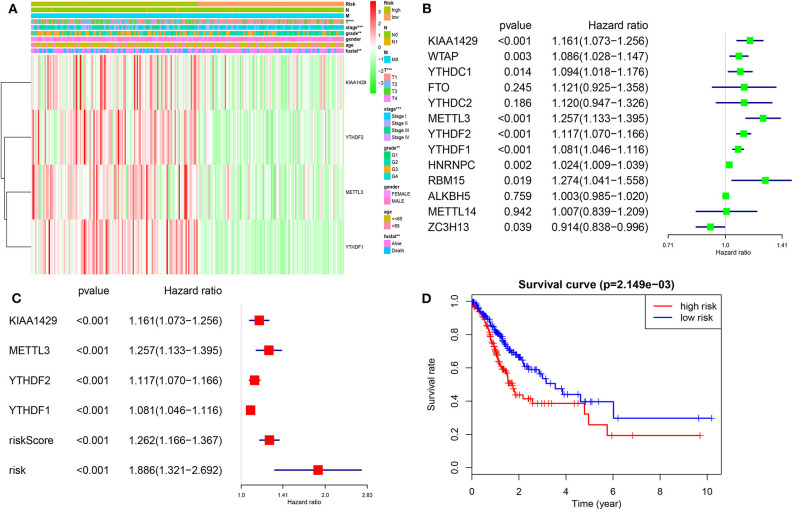
Interaction among m^6^A RNA methylation regulators and pathway analysis of HCC in cluster1/2 subgroups. **(A)** The m^6^A modification-related interactions among the 11-m^6^A RNA methylation regulators. **(B)** Spearman correlation analysis of the 13-m^6^A modification regulators. **(C–H)** GSEA revealed that genes with higher expression in cluster1 subgroup were enriched for hallmarks of malignant tumors.

The above results indicated that the clustering results were closely associated with the malignancy of the HCC. To better comprehend the interactions among these m^6^A RNA methylation regulators, which is significantly differentially expressed in tumor and normal tissues, we also analyzed the interaction (Figure 5A) and correlation (Figure 5B) among these gene regulators. WTAP, METTL3 and ALKBH5 seems to be the hub gene of the “writers” and “erasers,” and WTAP's interactions or co-expressions with RMB15, YTHDC1, YTHDF1, YTHDF2, YTHDC2, METTL3, KIAA1429, are validated by experimental data and by text mining in the String database (Figure 5A). In addition, METTL3's interactions or co-expressions with ALKBH5, WTAP, YTHDF1, RBM15, KIAA1429, YTHDC1, HNRNPC and FTO. The expression of WTAP was significantly associated with the “readers” of YTHDC1, YTHDF2, HNRNPC and YTHDF2 in HCC (Figure 5B). The expression of METTL3 was also significantly associated with “readers” of HNRNPC and YTHDC1 (*P* < 0.05). All these 11 differentially expressed m^6^A regulatory genes correlated each other, suggesting complicated mechanism underlying each interaction groups in HCC (Figure 3A), but the expressions of YTHDF2 were not significantly correlated with YTHDC2 and METTL14 in HCC (Figure 5B). Moreover, the expressions of all m^6^A regulatory genes were positively correlated with each other in HCC (Figure 5B). These findings were consistent with the expression levels of WTAP, METTL3, RBM15, KIAA1429, YTHDF2, YTHDF1, FTO, and ALKBH5 being positively correlated with the increasing HCC malignancy.

We then further applied principal component analysis (PCA) to compare the transcriptional profile between cluster1 and cluster2 groups. The findings revealed a clear difference between them (Figure 4C). We determined genes that were upregulated significantly (Score (d) for SAM > 8, fold change >2, and normalized *P* < 0.01) or downregulated [Score(d) for SAM < −8, fold change < 0.5, and normalized *P* < 0.01] in the cluster1 group, and then annotated their functions by pathway analysis for biological processes (Figures 5C–H).

The gene set enrichment analysis (GSEA) showed that cell cycle, notch signaling pathway, splicesome, ubiquitin mediated proteolysis and pathways in cancer were significantly associated with the cluster1 (Figures 5C–H, Table S9), while for cluster2, GSEA results showed metabolism-related pathways, including primary bile acid biosynthesis, drug metabolism and tryptophan metabolism (Table S10). All of these results showed that the two clusters determined by consensus clustering are closely associated with the carcinogenesis of HCC.

### Prognostic Value of m^6^A RNA Methylation Genes and a Risk Signature Built by Four-Selected m^6^A RNA Regulators

**Figure 6:**
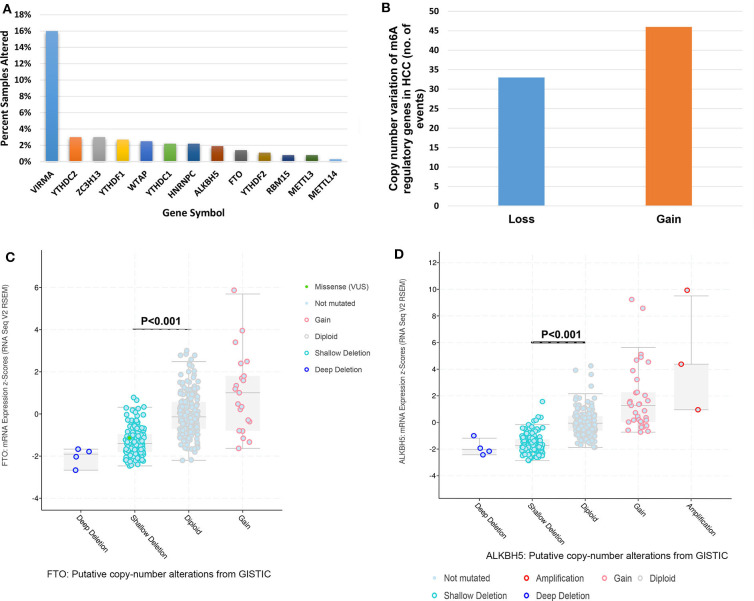
Risk signature with four m^6^A RNA methylation regulators. **(A)** Heatmap and clinicopathologic features of the two groups (risk low/high) defined by the m^6^A RNA methylation regulators consensus expression. **(B)** The process of building the signature containing 13 m^6^A RNA methylation regulators. The hazard ratios (HR), 95% confidence intervals (CI) calculated by univariate Cox regression. **(C)** The process of building the signature containing four m^6^A RNA methylation regulators calculated by machine learning model. **(D)** Kaplan–Meier overall survival (OS) curves for HCC patients according to m^6^Ascore. *P < 0.01; **P < 0.001; ***P < 0.0001.

We then investigated the prognostic value of m^6^A RNA regulators in HCC. We conducted a univariate Cox analysis on the gene expressions in the TCGA dataset (Figure 6). The results revealed that nine out of thirteen genes are significantly associated with OS (*P* < 0.05). Among these nine genes (Figure 6B), all the KIAA1429, WTAP, METLL3, YTHDC1, YTHDF2, YTHDF1, HNRNPC, RBM15, and ZC3H13 are risky genes with HR > 1. Furthermore, to better predict the HCC survivals with m^6^A RNA regulators, we applied the machine-learning model (Coxboost regression) to the nine prognosis-associated genes (Figure S3B) in the 75% TCGA dataset, which was used as a training cohort (Figure 6A). Four genes, including METTL3, KIAA1429, YTHDF2 and YTHDF1, were then selected to construct the risk signature according to the minimum criteria, and the coefficients derived from the Coxboost algorithm were applied to calculate the risk score for both the training dataset (75% TCGA) and the validation dataset (25% TCGA). To evaluate the prognostic value of the seven-m^6^A risk signature, we divided the HCC patients in the training set (*n* = 278) and validation set (*n* = 93) into low- and high-risk groups according to the median value of risk score and saw the significant differences in OS between the two clusters (both *P* = 0.002; Figure 6A). In addition, the prognostic value of m6Ascore are also prominent in another two GEO datasets (all *P* < 0.05; Figure S7). The heatmap shows the expression of the four selected m^6^A RNA regulators in the two groups including high- or low-risk HCC patients in the TCGA dataset (Figure 6A). The significant differences between the high and low risk groups with regards to metastasis (*P* < 0.001), tumor grade (*P* < 0.01) and clinical stage (*P* < 0.001) can be seen. The multivariate analysis showed the m^6^Ascore signature is an independent factor for OS in HCC patients (HR= 1.886, 95%CI 1.321–2.692, *P* < 0.001; Figure 6C). The ROC curve analysis showed that the m^6^Ascore can predict overall survival very well at 2,000 days (AUC = 0.70), 3-year survival (AUC = 0.69), cluster1/2 subgroups (AUC = 0.87) and TP53 mutations (AUC = 0.68; Figure S5). Furthermore, we explored whether m^6^Ascore could discriminate distinct survival stratified by TP53 mutations. The results showed m^6^Ascore can predict overall survival at 2,000 days well in TP53 mutations (AUC = 0.70) and TP53 wild-type (AUC = 0.61). These findings showed that the risk scores calculated by that signature could accurately predict HCC patient clinical outcomes and characteristics, especially for the cluster1/2 groups.

## Discussion

It remains a major challenge that identifying new molecular biomarkers tutors the evolvement of anti-HCC treatments. Our findings favored an evident association between genetic changes of m^6^A genes and TP53 mutation. One is confounding the other in predicting the prognosis of an HCC patient, which indicates that both might be complementary in the HCC pathogenesis or maintenance. The molecular biomarkers to identify tumor subtypes and patients prognosis still demand continuous refinement (30, 31). Regarding that, the m^6^A modification to mRNA owned wide biological functions; its impairment might be correlated with the progression of HCC. The current WHO classification emphasized epigenetic modifiers during the process of HCC clonal evolution as being mutated (30, 32, 33). Novel genetic subgroups embrace gene mutations that encode TP53 and epigenetic modifiers (27, 30, 32, 33).

Our research is the first to identify some clinical associations and effect of genetic changes influencing m^6^A genes in HCC. Although one previous study has showed that some m^6^A regulators, such as METTL3 and YTHDF1 were upregulated in HCC, and they were independent poor prognostic factors (34), we not only demonstrated that the expression of combined m^6^A regulators genes is also closely correlated with the prognosis of HCC, but also showed that a remarkable correlation between genetic changes of those m^6^A regulators as a whole group and the status of TP53 mutations (Table S1). More importantly, genetic changes of m^6^A regulators correlated with poorer clinical prognosis in HCC patients, even though this might be confounded by the unfavorable effects of the status of TP53 mutations on HCC survival (35). It has been revealed that the loss of METTL3 lead to alternative mRNA splicing and mRNA expression changes of more than 20 genes which are involved in the TP53 signaling pathway including MDM2, and P21 in HCC (4, 35). It is reasonable that genetic changes of m^6^A genes, TP53, or its regulator/downstream-molecular targets result in complementary pathways to the HCC pathogenesis. Hence, further studies in larger HCC cohorts could help confirm our results and spur future research into the functional effects of m^6^A RNA modification in HCC and its association with carcinogenesis pathways, especially for TP53 signaling.

Because our study has showed genetic changes of m^6^A genes, giving a deeper insight into their mechanism and link to HCC tumorigenesis pathways, the expressions of regulatory genes associated with clinical characteristics and its prognostic value have not been explored. Hence, we firstly determined two HCC subgroups, cluster1/2, by consensus clustering according to the mRNA expression of m^6^A regulators. The subgroups of cluster1/2 not only affect the HCC prognosis but also were closely associated with functional processes and cancer signaling pathways. Additionally, we established a novel prognostic risk signature with four m^6^A RNA regulators, stratifying the OS with HCC into high- and low-risk groups.

Because of the tumor tissue specificity of the “writers,” “erasers,” and “readers,” these genes involved in m^6^A dysregulation would be diverse in different cancers (32, 36). Among the m^6^A RNA methylation regulators, the writer METTL3 is often highly expressed in tumors and contributes to HCC tumorigenesis (19), which is consistent with our results; while METTL14, down regulated in HCC, acts as an unfavorable prognostic factor for HCC (8, 19). The reader YTHDF2 and the eraser FTO promotes cancer cell proliferation in pancreatic cancer and glioma (17, 37, 38). These results indicated that upregulation or downregulation of any m^6^A methylation regulators are associated with deregulated RNAs in cancers, and the same m^6^A regulators might have different biological functions in various cancers (9, 20, 37, 39).

In our HCC cohort, the frequency of genetic changes of the 13-m^6^A genes was much higher than that showed in AML (7), suggesting that the dysregulation of m^6^A may play a vital role in HCC carcinogenesis. Additionally, there was a high frequency of concurrent genetic changes of two regulatory genes, suggesting that m^6^A writer gene and reader gene might play a synergistic role during the process of RNA m^6^A modification (14, 20, 37, 40). In TP53-mutated samples, the expression of writer and reader genes, such as METTL3, RBM15, YTHDF1, and YTHDF2, were higher than wild-type samples, while the eraser genes, FTO and ALKBH5 were lower than the wild-type group (Figure S6). It indicates that the levels of m^6^A may correlated with the rate of TP53 mutations in HCC.

Unlike the CNVs in AML, most of the CNVs in writer and reader genes lead to the gain of function with up-regulation of the corresponding genes, while CNVs of the eraser genes were mainly gaining function resulting in down-regulation of the relevant genes. Regarding the opposite effect on m^6^A status for those two gene groups, these genetic changes increased the m^6^A level in HCC. Consistent with our findings, many researches on other cancers, like colorectal and pancreatic cancer (6, 20, 31, 36, 38, 40, 41) have also observed the up-regulated m^6^A level. This could be explained by the associations between m^6^A and cellular differentiation pathways controlling cancer stem cell fate (29, 31, 42).

We also comprehensively analyzed the expression of all m^6^A RNA regulators in HCC with different clinical characteristics. As an m^6^A methylation writer, the expression of METLL3 was increased in tumor group, higher tumor grade and stage. WTAP expression was significantly increased in higher-grade and metastasis. For the m^6^A methylation readers, the expression of HNRNPC, YTHDF1, and YTHDF2 was also significantly increased in higher tumor grade and stage. Interestingly, the expression of FTO was decreased in no metastasis, lower tumor grade and stage. Taken together, the expression of m^6^A RNA regulators is closely correlated with malignant clinical characteristics in HCC. These results are also helpful for establishing new therapeutic methods through characterizing the expression of individual m^6^A regulators in HCC, as chemicals targeting m^6^A methylation are regarded as a novel method of cancer treatment (43).

Whether the expression level of m^6^A RNA methylation genes could be applied as a prognostic biomarker plays a vital role in the research of cancers. In this study, our HCC prognostic signature derived using four m^6^A RNA methylation regulators was found to stratify the OS for TP53 mutations and cluster1/2 subgroups. A similar scenario was also seen in the multivariate Cox regression analysis. This might be caused by the strong association between the m^6^Ascore and TP53 status.

## Conclusions

Overall, our results systematically revealed the genetic changes, mRNA expression, potential biological function, and clinical prognostic value of m^6^A RNA methylation regulators in HCC. We observed a remarkable relationship between the genetic changes and different expressions lead to increased m^6^A level and poorer clinical prognosis. It is reasonable that genetic changes of m^6^A modifiers, TP53, or its regulator/downstream spots contribute in complementary pathways to the pathogenesis of HCC. Additionally, the expressions of m^6^A RNA methylation regulators are associated with the increased expression levels of genes significantly enriched in the biological processes and cancer signaling pathways that facilitate the HCC malignant progression. Finally, our research confers vital evidence for future investigation of the role of mRNA m^6^A methylation in HCC.

## Data Availability Statement

The datasets presented in this study can be found in online repositories. The names of the repository/repositories and accession number(s) can be found in the article/Supplementary Material.

## Ethics Statement

The studies involving human participants were reviewed and approved by TCGA database. The patients/participants provided their written informed consent to participate in this study.

## Author Contributions

G-QZ, LY, Y-JZ, J-XD, Y-MW, and ZD designed the study. G-QZ, Y-MW, J-XD collected and downloaded data. LY, Y-JZ did the statistical analyses. Y-HS and S-SD prepared figures. G-QZ, LY, JF, JZ, S-SD, and Y-HS reviewed the results, interpreted data, and wrote the manuscript. All authors have made an intellectual contribution to the manuscript and approved the submission.

## Conflict of Interest

The authors declare that the research was conducted in the absence of any commercial or financial relationships that could be construed as a potential conflict of interest.

## Supplementary Material

Figure S1 Genetic alteration spectrum of m^6^A regulatory genes for HCC.

Figure S2 Relationships between putative copy-number alterations and m^6^A genes' mRNA expression.

Figure S3 Gene expression of m^6^A regulatory enzymes and booting step by machine learning model.

Figure S4 Consensus clustering cumulative distribution function (CDF) for *k* = 2–10.

Figure S5 ROC curves with AUCs of prognostic predictors built by m^6^Ascore calculated by m^6^A regulatory genes.

Figure S6 Gene expression of m^6^A regulatory genes between TP53 mutated and wild-type sample.

Figure S7 Validation of prognostic value of m^6^A score in two GEO datasets.

Table S1 Clinical and molecular characteristics of TCGA HCC patients according to the mutation and/or copy number variation status of genes encoding m^6^A regulatory enzymes.

Table S2 Genetic alterations of 13 m^6^A regulatory genes in HCC.

Table S3 HCC samples with a point mutation, deep deletion, amplification, shallow deletion and/or copy number gain of one or more genes encoding m^6^A regulatory enzymes. ^*^Examples of potentially synergistic changes that may increase RNA m^6^A levels.

Table S4 Clinical and molecular characteristics of HCC patients with an amplification or copy number gain of the gene encoding an m^6^A writer, VIRMA.

Table S5 Univariate analysis for clinicopathological and molecular features.

Table S6 Subgroup analysis for alterations of m^6^A regulatory genes as a group associated with overall survival.

Table S7 Univariate analysis for overall survival of patients stratified by the status of m^6^A regulatory gene alterations in addition to (A) TP53, (B) TERT, and (C) ARID1A mutation status.

Table S8 Multivariate analysis for overall survival in HCC patients.

Table S9 Pathway analysis for cluster1 in HCC.

Table S10 Pathway analysis for cluster2 in HCC.

## Abbreviations

HCC: hepatocellular carcinoma
OS: overall survival
CI: confidence interval
HR: hazard risk
m^6^A: N6-methyladenosine RNA methylation
CNV: copy number variations
METTL3: methyltransferase like 3
WTAP: WT1-associated protein
RBM15: RNA binding motif protein 15
ZC3H13: zinc finger CCCH domain-containing protein 13
YTHDC1: YTH domain-containing 1
YTHDF1: YTH N6-methyl-adenosine RNA binding protein 1
HNRNPC: and heterogeneous nuclear ribonucleoprotein C
FTO: fat mass- and obesity-associated protein
ALKBH5: α-ketoglutarate-dependent dioxygenase alkB
TCGA: The Cancer Genome Atlas
GISTIC: Genomic Identification of Significant Targets in Cancer algorithm
DFS: disease-free survival
PCA: principal component analysis
GSEA: gene set enrichment analysis.
