# Supplementary figures and images for: Genetic Alterations and Transcriptional Expression of m6A RNA Methylation Regulators Drive a Malignant Phenotype and Have Clinical Prognostic Impact in Hepatocellular Carcinoma
Source: Front Oncol. 2020 Jul 21;10:900. doi: 10.3389/fonc.2020.00900 (PMC7396691; doi:10.3389/fonc.2020.00900)

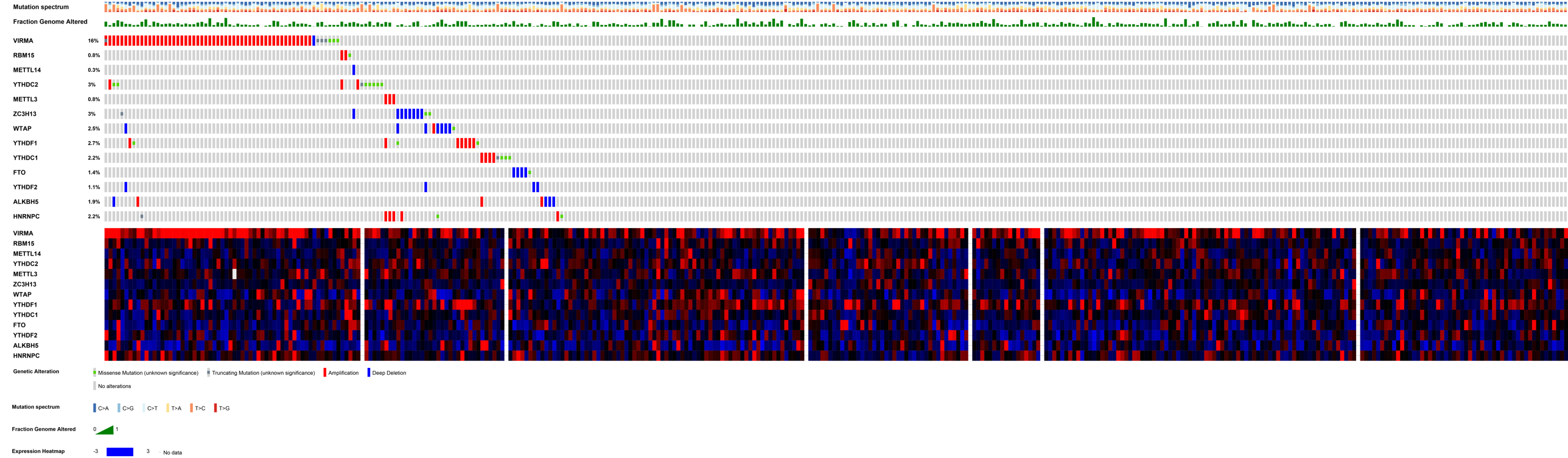

Supplement: Figure S1 — Genetic alteration spectrum of m6A regulatory genes for HCC. [file Image_1.TIF]

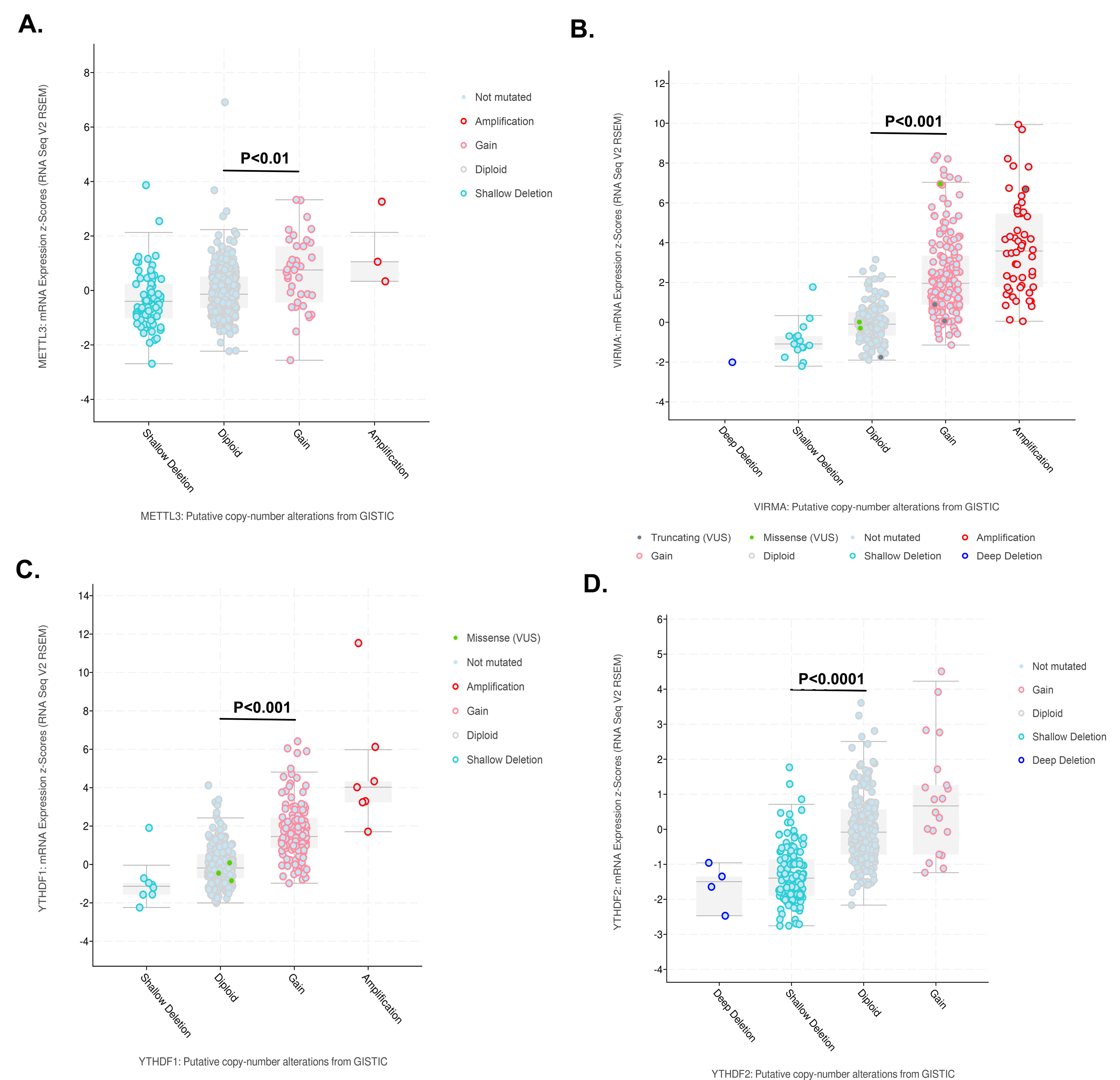

Supplement: Figure S2 — Relationships between putative copy-number alterations and m6A genes' mRNA expression. [file Image_2.TIF]

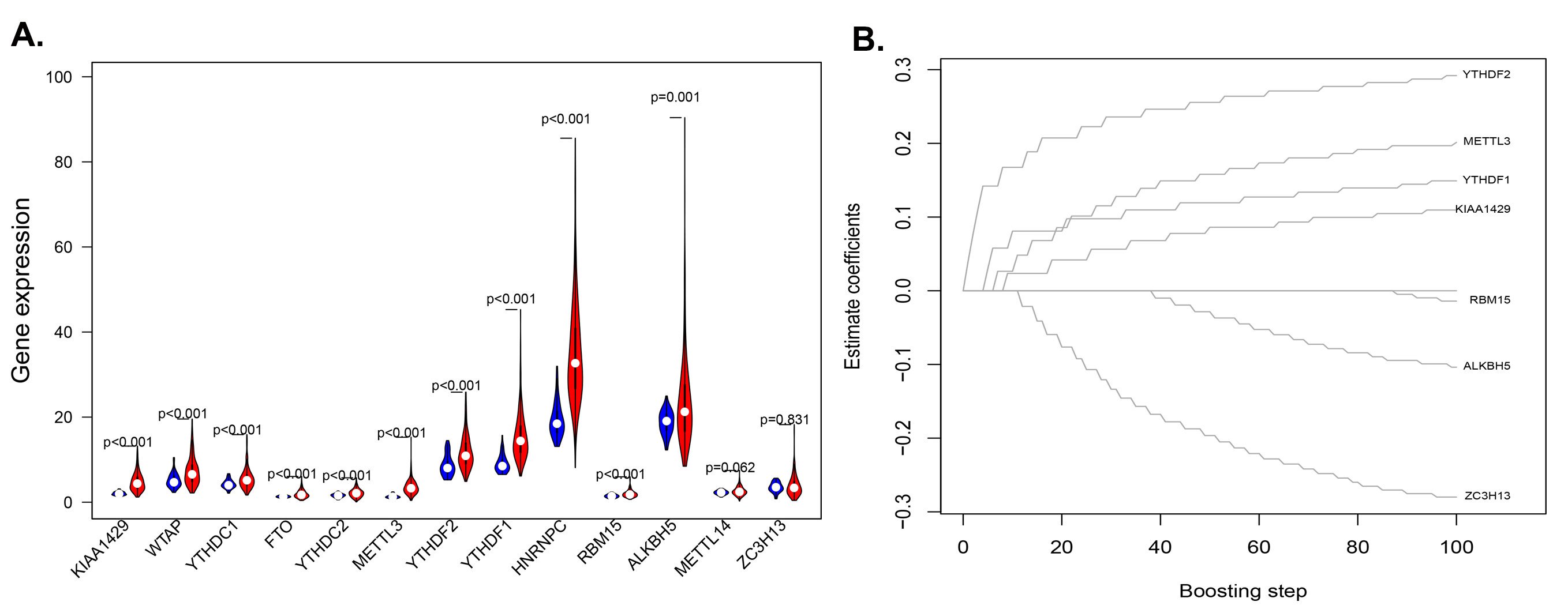

Supplement: Figure S3 — Gene expression of m6A regulatory enzymes and booting step by machine learning model. [file Image_3.TIF]

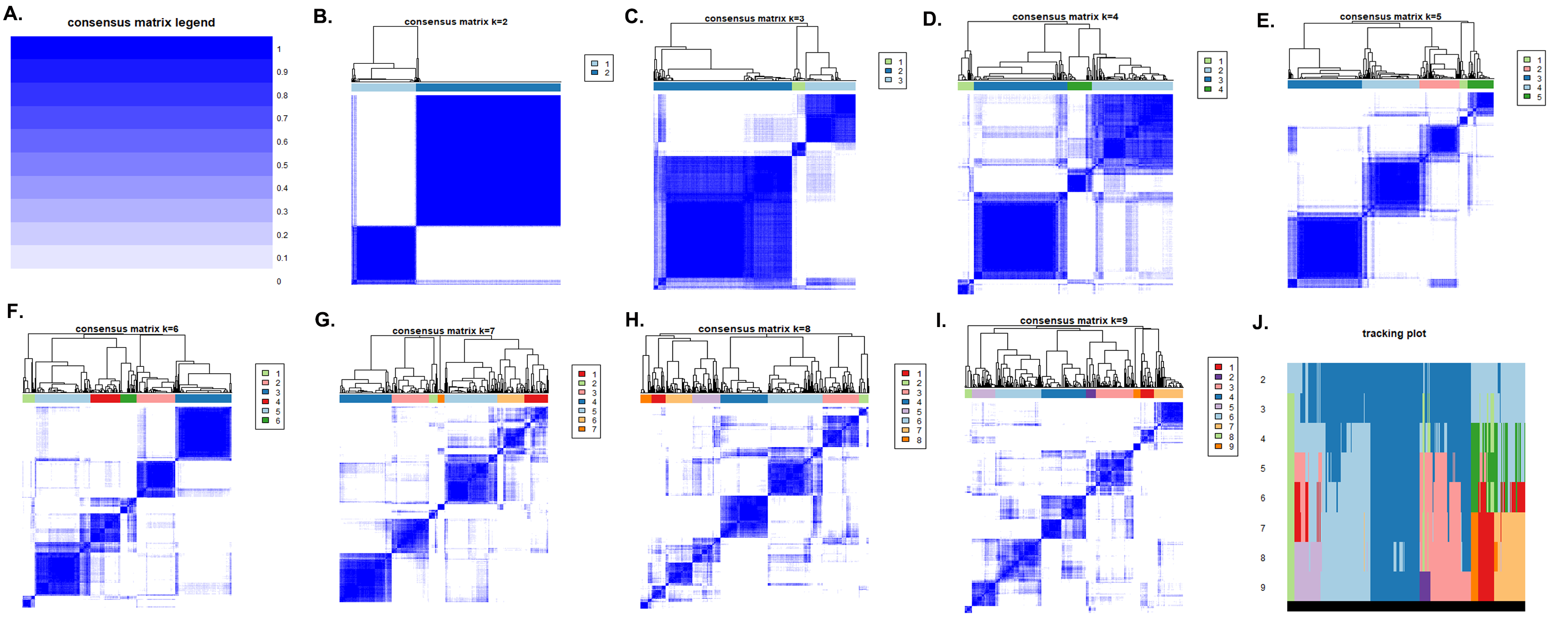

Supplement: Figure S4 — Consensus clustering cumulative distribution function (CDF) for k = 2–10. [file Image_4.TIF]

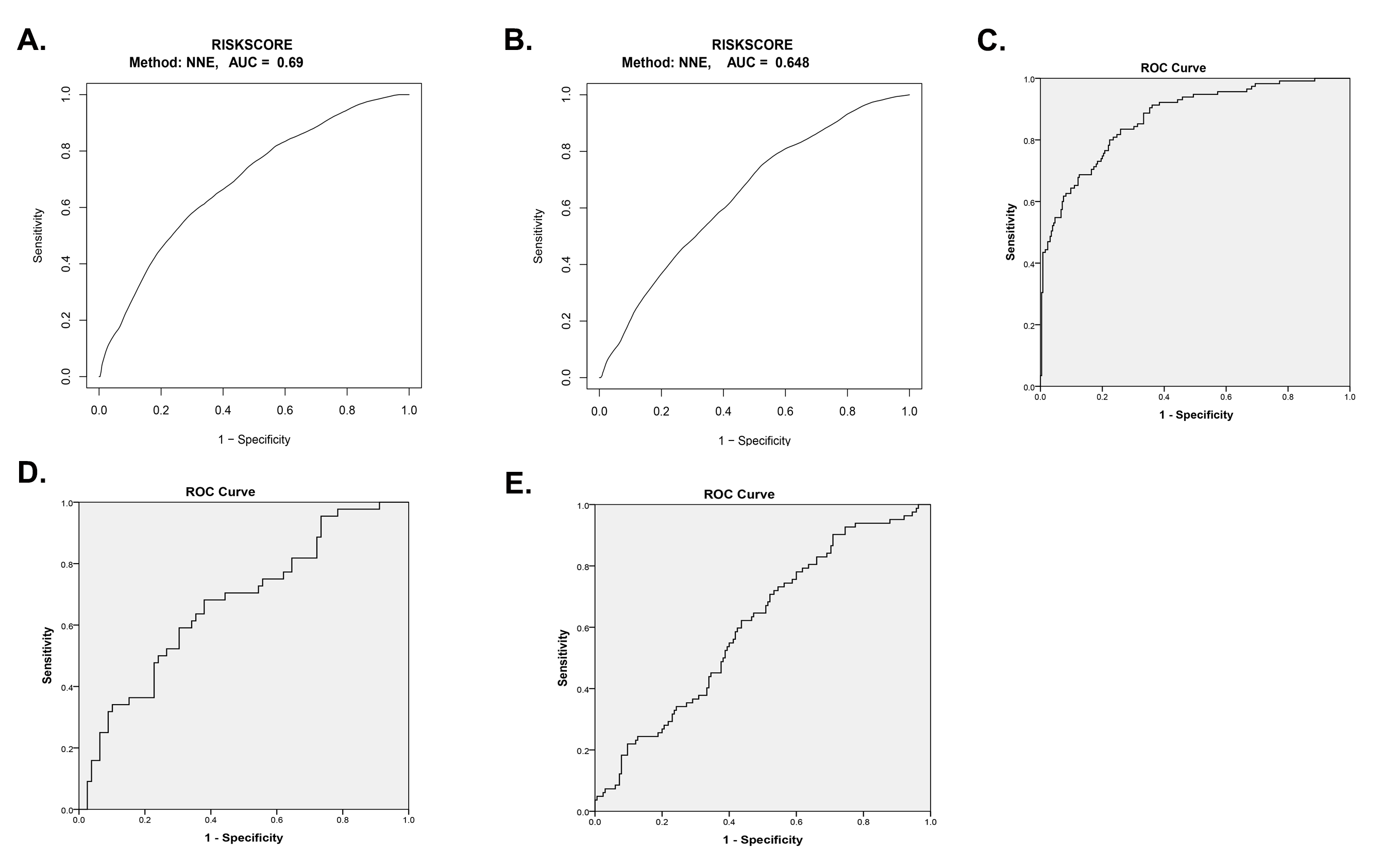

Supplement: Figure S5 — ROC curves with AUCs of prognostic predictors built by m6Ascore calculated by m6A regulatory genes. [file Image_5.TIF]

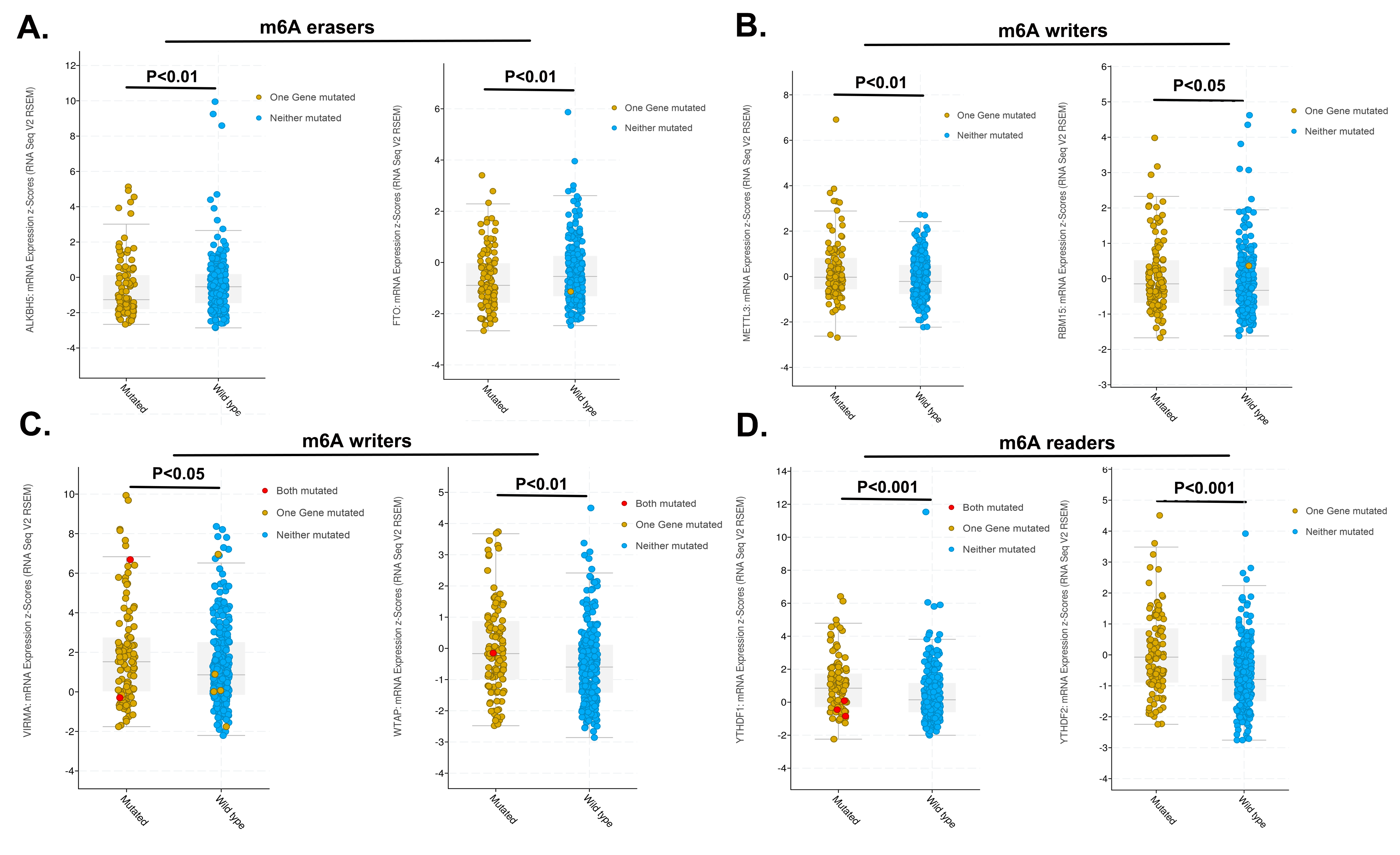

Supplement: Figure S6 — Gene expression of m6A regulatory genes between TP53 mutated and wild-type sample. [file Image_6.TIF]

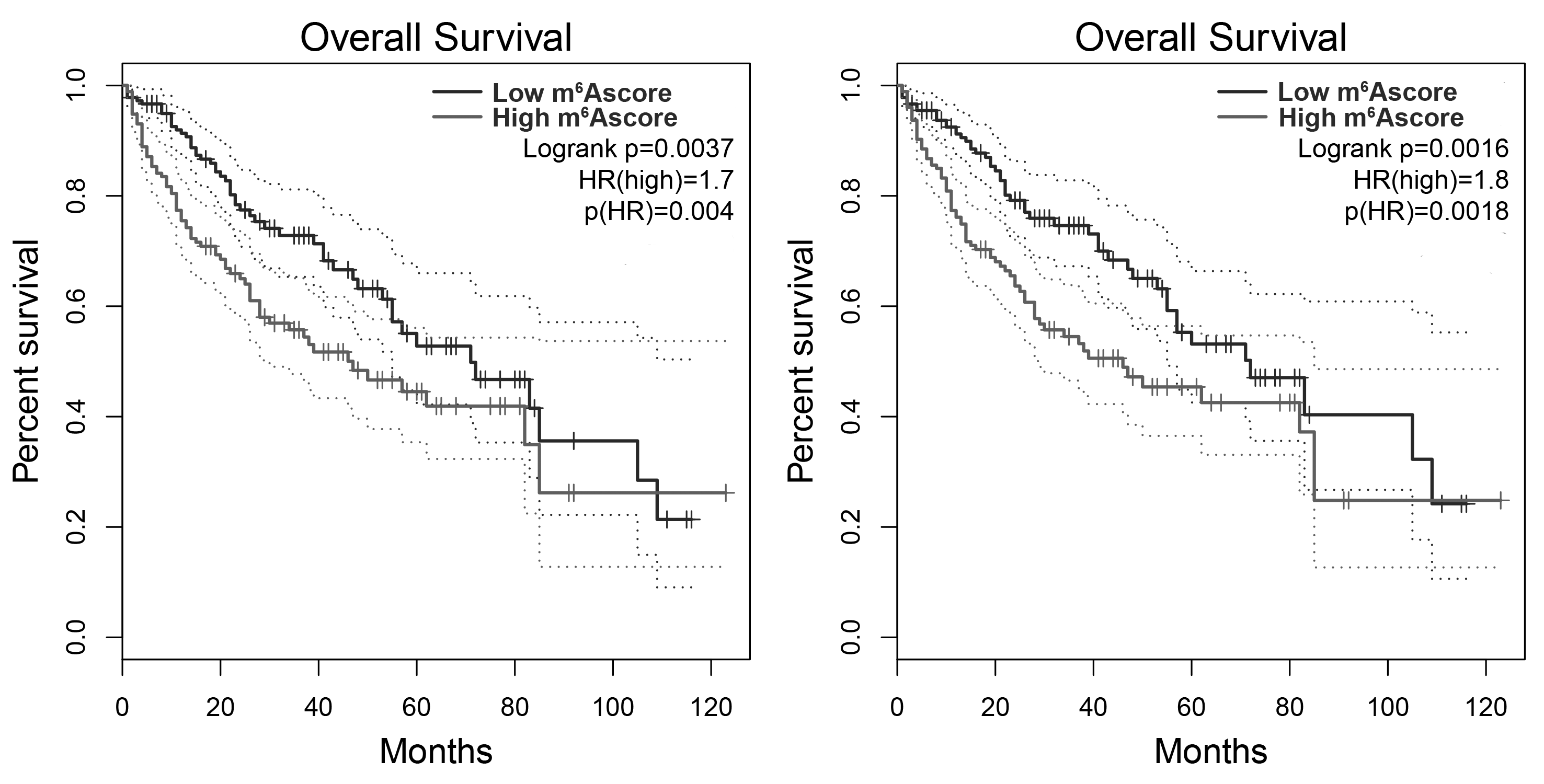

Supplement: Figure S7 — Validation of prognostic value of m6A score in two GEO datasets. [file Image_7.TIF]
