# Supplementary material for: Genetic Alterations and Transcriptional Expression of m6A RNA Methylation Regulators Drive a Malignant Phenotype and Have Clinical Prognostic Impact in Hepatocellular Carcinoma
Source: Front Oncol. 2020 Jul 21;10:900. doi: 10.3389/fonc.2020.00900 (PMC7396691; doi:10.3389/fonc.2020.00900)
Supplement: Table S1 — Clinical and molecular characteristics of TCGA HCC patients according to the mutation and/or copy number variation status of genes encoding m6A regulatory enzymes. [file Table_1.DOCX]

**Table S1.** Clinical and molecular characteristics of TCGA HCC patients according to the mutation and/or copy number variation status of genes encoding m6A regulatory enzymes.

|  | Mutation and/or CNV | | | Mutation only | | | CNV only | | |
| --- | --- | --- | --- | --- | --- | --- | --- | --- | --- |
|  | No | Yes | P-value | No | Yes | P-value | No | Yes | P-value |
| No. of patients | 252 | 115 |  | 336 | 31 |  | 281 | 84 |  |
| BMI (kg/m^2^) | 25.8 ± 6.4 | 26.9 ± 11.8 | 0.242 | 26.2 ± 8.8 | 25.0 ± 4.5 | 0.459 | 25.7 ± 6.3 | 27.7 ± 13.6 | 0.074 |
| Albumin (mg/dl) | 4.4 ± 5.0 | 4.1 ± 4.9 | **0.038** | 4.4 ± 5.2 | 3.7 ± 1.0 | 0.529 | 4.4 ± 4.8 | 4.2 ± 5.6 | 0.861 |
| Bilirubin (mg/dl) | 1.6 ± 6.2 | 1.0 ± 1.3 | 0.408 | 1.5 ± 5.4 | 1.0 ± 0.7 | 0.727 | 1.5 ± 5.9 | 1.0 ± 1.5 | 0.491 |
| Creatinine (mg/dl) | 1.3 ± 1.8 | 1.0 ± 0.5 | 0.116 | 1.2 ± 1.6 | 1.0 ± 0.3 | 0.427 | 1.3 ± 1.7 | 1.0 ± 0.5 | 0.225 |
| Platelet count (/10^3^) | 223.0 ± 114.7 | 217.5 ± 96.0 | 0.756 | 223.1 ± 109.9 | 192.6 ± 95.5 | 0.393 | 220.8 ± 113.3 | 223.2 ± 96.3 | 0.897 |
| Prothrombin time (s) | 3.5 ± 4.1 | 4.5 ± 4.9 | 0.068 | 3.8 ± 4.4 | 3.9 ± 4.6 | 0.959 | 3.5 ± 4.1 | 4.7 ± 5.1 | 0.183 |
| Sex |  |  | 0.158 |  |  | 0.189 |  |  | 0.189 |
| Female | 87 (34.9%) | 31 (27.4%) |  | 112 (33.5%) | 6 (21.4%) |  | 112 (33.5%) | 6 (21.4%) |  |
| Male | 162 (65.1%) | 82 (72.6%) |  | 222 (66.5%) | 22 (78.6%) |  | 222 (66.5%) | 22 (78.6%) |  |
| T |  |  | 0.183 |  |  | 0.543 |  |  | 0.543 |
| 0 | 114 (45.4%) | 62 (55.9%) |  | 161 (48.1%) | 15 (55.6%) |  | 161 (48.1%) | 15 (55.6%) |  |
| 1 | 71 (28.3%) | 23 (20.7%) |  | 86 (25.7%) | 8 (29.6%) |  | 86 (25.7%) | 8 (29.6%) |  |
| 2 | 55 (21.9%) | 24 (21.6%) |  | 75 (22.4%) | 4 (14.8%) |  | 75 (22.4%) | 4 (14.8%) |  |
| 3 | 11 (4.4%) | 2 (1.8%) |  | 13 (3.9%) | 0 (0.0%) |  | 13 (3.9%) | 0 (0.0%) |  |
| N |  |  | 0.799 |  |  | 0.577 |  |  | 0.577 |
| 0 | 172 (98.3%) | 77 (98.7%) |  | 231 (98.3%) | 18 (100.0%) |  | 231 (98.3%) | 18 (100.0%) |  |
| 1 | 3 (1.7%) | 1 (1.3%) |  | 4 (1.7%) | 0 (0.0%) |  | 4 (1.7%) | 0 (0.0%) |  |
| M |  |  | 0.799 |  |  | 0.535 |  |  | 0.535 |
| 0 | 181 (98.4%) | 81 (98.8%) |  | 239 (98.4%) | 23 (100.0%) |  | 239 (98.4%) | 23 (100.0%) |  |
| 1 | 3 (1.6%) | 1 (1.2%) |  | 4 (1.6%) | 0 (0.0%) |  | 4 (1.6%) | 0 (0.0%) |  |
| AJCC. stage |  |  | **0.0043** |  |  | **0.030** |  |  | **0.004** |
| I | 109 (46.4%) | 57 (53.8%) |  | 151 (48.1%) | 15 (55.6%) |  | 151 (48.1%) | 15 (55.6%) |  |
| II | 63 (26.8%) | 23 (21.7%) |  | 78 (24.8%) | 8 (29.6%) |  | 78 (24.8%) | 8 (29.6%) |  |
| III | 53 (22.6%) | 24 (22.6%) |  | 73 (23.2%) | 4 (14.8%) |  | 73 (23.2%) | 4 (14.8%) |  |
| IV | 10 (4.3%) | 2 (1.9%) |  | 12 (3.8%) | 0 (0.0%) |  | 12 (3.8%) | 0 (0.0%) |  |
| Tumor grade |  |  | 0.431 |  |  | 0.729 |  |  | 0.729 |
| I | 33 (13.3%) | 20 (18.0%) |  | 49 (14.7%) | 4 (14.8%) |  | 49 (14.7%) | 4 (14.8%) |  |
| II | 126 (50.6%) | 50 (45.0%) |  | 161 (48.3%) | 15 (55.6%) |  | 161 (48.3%) | 15 (55.6%) |  |
| III/IV | 90 (36.1%) | 41 (36.9%) |  | 123 (36.9%) | 8 (29.6%) |  | 123 (36.9%) | 8 (29.6%) |  |
| New recurrence site |  |  | 0.395 |  |  | 0.313 |  |  | 0.914 |
| liver | 76 (74.5%) | 38 (74.5%) |  | 84 (75.0%) | 30 (73.2%) |  | 106 (74.1%) | 8 (80.0%) |  |
| lung | 14 (13.7%) | 4 (7.8%) |  | 15 (13.4%) | 3 (7.3%) |  | 17 (11.9%) | 1 (10.0%) |  |
| others | 12 (11.8%) | 9 (17.6%) |  | 13 (11.6%) | 8 (19.5%) |  | 20 (14.0%) | 1 (10.0%) |  |
| Embolization performed |  |  | 0.635 |  |  | 0.363 |  |  | 0.363 |
| No | 7 (25.9%) | 4 (33.3%) |  | 11 (29.7%) | 0 (0.0%) |  | 11 (29.7%) | 0 (0.0%) |  |
| Yes | 20 (74.1%) | 8 (66.7%) |  | 26 (70.3%) | 2 (100.0%) |  | 26 (70.3%) | 2 (100.0%) |  |
| Child-Pugh grade |  |  | 0.548 |  |  | 0.847 |  |  | 0.847 |
| A | 129 (92.8%) | 56 (90.3%) |  | 171 (91.9%) | 14 (93.3%) |  | 171 (91.9%) | 14 (93.3%) |  |
| B | 10 (7.2%) | 6 (9.7%) |  | 15 (8.1%) | 1 (6.7%) |  | 15 (8.1%) | 1 (6.7%) |  |
| Vital status |  |  | 0.961 |  |  | 0.863 |  |  | 0.863 |
| HBV-affected | 64 (85.3%) | 24 (85.7%) |  | 81 (85.3%) | 7 (87.5%) |  | 81 (85.3%) | 7 (87.5%) |  |
| HCV-affected | 11 (14.7%) | 4 (14.3%) |  | 14 (14.7%) | 1 (12.5%) |  | 14 (14.7%) | 1 (12.5%) |  |
| TP53 |  |  | **0.012** |  |  | **0.015** |  |  | 0.781 |
| Wild-type | 215 (85.3%) | 69 (60.0%) |  | 232 (68.8%) | 13 (46.4%) |  | 189 (67.5%) | 56 (65.9%) |  |
| Mutation | 37 (14.7%) | 46 (40%) |  | 105 (31.2%) | 15 (53.6%) |  | 91 (32.5%) | 29 (34.1%) |  |
| TERT |  |  | **0.018** |  |  | 0.949 |  |  | **0.011** |
| Wild-type | 240 (95.2%) | 100 (88.5%) |  | 314 (93.2%) | 26 (92.9%) |  | 266 (95.0%) | 74 (87.1%) |  |
| Mutation | 12 (4.8%) | 13 (11.5%) |  | 23 (6.8%) | 2 (7.1%) |  | 14 (5.0%) | 11 (12.9%) |  |
| CTNNB1 |  |  | 0.173 |  |  | 0.132 |  |  | 0.588 |
| Wild-type | 189 (75.0%) | 77 (68.1%) |  | 249 (73.9%) | 17 (60.7%) |  | 206 (73.6%) | 60 (70.6%) |  |
| Mutation | 63 (25.0%) | 36 (31.9%) |  | 88 (26.1%) | 11 (39.3%) |  | 74 (26.4%) | 25 (29.4%) |  |
| CCND1 |  |  | 0.571 |  |  | 0.529 |  |  | 0.823 |
| Wild-type | 234 (92.9%) | 103 (91.2%) |  | 312 (92.6%) | 25 (89.3%) |  | 259 (92.5%) | 78 (91.8%) |  |
| Mutation | 18 (7.1%) | 10 (8.8%) |  | 25 (7.4%) | 3 (10.7%) |  | 21 (7.5%) | 7 (8.2%) |  |
| AXIN1 |  |  | 0.571 |  |  | 0.529 |  |  | 0.823 |
| Wild-type | 234 (92.9%) | 103 (91.2%) |  | 312 (92.6%) | 25 (89.3%) |  | 259 (92.5%) | 78 (91.8%) |  |
| Mutation | 18 (7.1%) | 10 (8.8%) |  | 25 (7.4%) | 3 (10.7%) |  | 21 (7.5%) | 7 (8.2%) |  |
| ARID2 |  |  | 0.056 |  |  | 0.105 |  |  | 0.286 |
| Wild-type | 239 (94.8%) | 101 (89.4%) |  | 316 (93.8%) | 24 (85.7%) |  | 263 (93.9%) | 77 (90.6%) |  |
| Mutation | 13 (5.2%) | 12 (10.6%) |  | 21 (6.2%) | 4 (14.3%) |  | 17 (6.1%) | 8 (9.4%) |  |
| ARID1A |  |  | **0.047** |  |  | **0.027** |  |  | **0.0037** |
| Wild-type | 233 (92.5%) | 97 (85.8%) |  | 308 (91.4%) | 22 (78.6%) |  | 255 (91.1%) | 67(88.2%) |  |
| Mutation | 19 (7.5%) | 16 (14.2%) |  | 29 (8.6%) | 6 (21.4%) |  | 17(6.0%) | 18 (21.4%) |  |

Significant P values are in bold; CNV: copy number variation
