# Supplementary material for: Genetic Alterations and Transcriptional Expression of m6A RNA Methylation Regulators Drive a Malignant Phenotype and Have Clinical Prognostic Impact in Hepatocellular Carcinoma
Source: Front Oncol. 2020 Jul 21;10:900. doi: 10.3389/fonc.2020.00900 (PMC7396691; doi:10.3389/fonc.2020.00900)
Supplement: Table S4 — Clinical and molecular characteristics of HCC patients with an amplification or copy number gain of the gene encoding an m6A writer, VIRMA. [file Table_4.DOCX]

**Table S4.** Clinical and molecular characteristics of HCC patients with an amplification or copy number gain of the gene encoding an m^6^A writer, *VIRMA*.

|  | Amplification or copy number gain of *VIRMA* | | |
| --- | --- | --- | --- |
|  | No | Yes | P-value |
| No of patients | 306 | 59 |  |
| BMI | 25.8 ± 6.4 | 27.8 ± 15.6 | 0.130 |
| Albumin (mg/dl) | 4.3 ± 4.5 | 4.5 ± 6.9 | 0.771 |
| Bilirubin (mg/dl) | 1.5 ± 5.6 | 1.2 ± 1.8 | 0.742 |
| Creatinine (mg/dl) | 1.3 ± 1.6 | 1.0 ± 0.6 | 0.412 |
| Platelet count (/10^3^) | 221.0 ± 113.4 | 223.4 ± 82.2 | 0.919 |
| Prothrombin time (s) | 3.7 ± 4.3 | 4.8 ± 5.1 | 0.276 |
| TP53 |  |  | **0.0431** |
| Wild-type | 208 (68.0%) | 37 (62.7%) |  |
| Mutation | 90 (29.4.0%) | 30 (50.8%) |  |
| TERT |  |  | **0.027** |
| Wild-type | 287 (93.8%) | 53 (89.8%) |  |
| Mutation | 10 (3.3%) | 16 (27.2%) |  |
| CTNNB1 |  |  | 0.055 |
| Wild-type | 229 (74.8%) | 37 (62.7%) |  |
| Mutation | 77 (25.2%) | 22 (37.3%) |  |
| CCND1 |  |  | 0.415 |
| Wild-type | 281 (91.8%) | 56 (94.9%) |  |
| Mutation | 25 (8.2%) | 3 (5.1%) |  |
| AXIN1 |  |  | 0.779 |
| Wild-type | 282 (92.2%) | 55 (93.2%) |  |
| Mutation | 24 (7.8%) | 4 (6.8%) |  |
| ARID2 |  |  | 0.096 |
| Wild-type | 288 (94.1%) | 52 (88.1%) |  |
| Mutation | 18 (5.9%) | 7 (11.9%) |  |
| ARID1A |  |  | 0.869 |
| Wild-type | 277 (90.5%) | 53 (89.8%) |  |
| Mutation | 29 (9.5%) | 6 (10.2%) |  |
| Sex |  |  | 0.112 |
| Female | 104 (34.3%) | 14 (23.7%) |  |
| Male | 199 (65.7%) | 45 (76.3%) |  |
| T |  |  | 0.718 |
| 0 | 145 (47.7%) | 31 (53.4%) |  |
| 1 | 81 (26.6%) | 13 (22.4%) |  |
| 2 | 66 (21.7%) | 13 (22.4%) |  |
| 3 | 12 (3.9%) | 1 (1.7%) |  |
| N |  |  | 0.649 |
| 0 | 208 (98.6%) | 41 (97.6%) |  |
| 1 | 3 (1.4%) | 1 (2.4%) |  |
| M |  |  | 0.646 |
| 0 | 219 (98.6%) | 43 (97.7%) |  |
| 1 | 3 (1.4%) | 1 (2.3%) |  |
| AJCC. stage |  |  | **0.008** |
| I | 137 (48.2%) | 29 (50.9%) |  |
| II | 73 (25.7%) | 13 (22.8%) |  |
| III | 63 (22.2%) | 14 (24.6%) |  |
| IV | 11 (3.9%) | 1 (1.8%) |  |
| Tumor grade |  |  | 0.504 |
| I | 45 (14.9%) | 8 (13.8%) |  |
| II | 151 (50.0%) | 25 (43.1%) |  |
| III/IV | 106 (35.1%) | 25 (43.1%) |  |
| New recurrence site |  |  | 0.885 |
| liver | 97 (75.2%) | 17 (70.8%) |  |
| lung | 15 (11.6%) | 3 (12.5%) |  |
| others | 17 (13.2%) | 4 (16.7%) |  |
| Embolization performed |  |  | 0.342 |
| No | 8 (25.0%) | 3 (42.9%) |  |
| Yes | 24 (75.0%) | 4 (57.1%) |  |
| Child-Pugh grade |  |  | 0.081 |
| A | 158 (93.5%) | 27 (84.4%) |  |
| B | 11 (6.5%) | 5 (15.6%) |  |
| Vital status |  |  | 0.352 |
| HBV-affected | 78 (86.7%) | 10 (76.9%) |  |
| HCV-affected | 12 (13.3%) | 3 (23.1%) |  |

Significant P values are in bold;
