# Supplementary material for: Genetic Alterations and Transcriptional Expression of m6A RNA Methylation Regulators Drive a Malignant Phenotype and Have Clinical Prognostic Impact in Hepatocellular Carcinoma
Source: Front Oncol. 2020 Jul 21;10:900. doi: 10.3389/fonc.2020.00900 (PMC7396691; doi:10.3389/fonc.2020.00900)
Supplement: Table S5 — Univariate analysis for clinicopathological and molecular features. [file Table_5.DOCX]

**Table S5.** Univariate analysis for clinicopathological and molecular features.

|  | Univariate analysis |
| --- | --- |
|  |  |
| Exposure | Hazard ratio (95% CI) P value |
| BMI | 1.4 (0.3, 3.1) 0.632 |
| Albumin (mg/dl) | 1.1 (0.8, 1.4) 0.213 |
| Bilirubin (mg/dl) | 0.8 (0.5, 1.2) 0.255 |
| Creatinine (mg/dl) | 1.5 (0.9, 4.2) 0.721 |
| Platelet count (/10^3^) | 1.7 (0.2, 3.5) 0.261 |
| Prothrombin time (s) | 2.3 (0.6, 8.3) 0.831 |
| T |  |
| 0 | 1.0 |
| 1 | 1.4 (0.9, 2.2) 0.184 |
| 2 | **2.6 (1.7, 3.9) <0.001** |
| 3 | **5.1 (2.5, 10.2) <0.001** |
| N |  |
| 0 | 1.0 |
| 1 | 2.0 (0.5, 8.1) 0.341 |
| M |  |
| 0 | 1.0 |
| 1 | 3.9 (1.2, 12.5) 0.021 |
| Tumor grade |  |
| I | 1.0 |
| II | 1.2 (0.7, 2.0) 0.588 |
| III/IV | 1.2 (0.7, 2.1) 0.477 |
| Embolization performed |  |
| No | 1.0 |
| Yes | 2.9 (0.9, 10.0) 0.088 |
| Child-Pugh grade |  |
| A | 1.0 |
| B | 1.7 (0.7, 4.4) 0.250 |
| Vital status |  |
| HBV-affected | 1.0 |
| HCV-affected | 0.7 (0.3, 1.6) 0.370 |
| AJCC. stage |  |
| I | 1.0 |
| II | 1.4 (0.8, 2.2) 0.227 |
| III | **2.4 (1.6, 3.8) <0.001** |
| IV | **5.3 (2.5, 11.5) <0.001** |
| TP53 |  |
| Wild-type | 1.0 |
| Mutation | **1.2 (1.0, 1.8) 0.026** |
| TERT |  |
| Wild-type | 1.0 |
| Mutation | 0.9 (0.4, 1.9) 0.804 |
| ARID2 |  |
| Wild-type | 1.0 |
| Mutation | 0.8 (0.4, 1.6) 0.477 |
| ARID1A |  |
| Wild-type | 1.0 |
| Mutation | **1.9 (1.2, 3.2) 0.011** |
| m^6^A genes status |  |
| Without genetic alterations | 1.0 |
| With mutations and/or CNVs | 1.7 (0.5, 1.0) 0.177 |

Significant P values are in bold;
