# Supplementary material for: Genetic Alterations and Transcriptional Expression of m6A RNA Methylation Regulators Drive a Malignant Phenotype and Have Clinical Prognostic Impact in Hepatocellular Carcinoma
Source: Front Oncol. 2020 Jul 21;10:900. doi: 10.3389/fonc.2020.00900 (PMC7396691; doi:10.3389/fonc.2020.00900)
Supplement: Table S6 — Subgroup analysis for alterations of m6A regulatory genes as a group associated with overall survival. [file Table_6.docx]

**Table S6.** Subgroup analysis for alterations of m6A regulatory genes as a group associated with overall survival.

| Parameters and model | Hazard ratio (CI) P-value | P for interaction |
| --- | --- | --- |
| T |  | 0.120 |
| 0 | 1.1 (0.7, 2.1) 0.213 |  |
| 1 | 1.4 (0.9, 2.2) 0.184 |  |
| 2 | 2.1 (0.7, 3.9) 0.441 |  |
| 3 | 3.2 (0.3, 10.2) 0.513 |  |
| N |  | 0.311 |
| 0 | 0.7 (0.3, 2.2) 0.123 |  |
| 1 | 2.1 (0.5, 4.1) 0.141 |  |
| Tumor grade |  | 0.971 |
| I | 1.1 (0.5, 2.5) 0.621 |  |
| II | 1.4 (0.7, 2.0) 0.588 |  |
| III/IV | 1.1 (0.3, 4.1) 0.477 |  |
| Embolization performed |  |  |
| No | 2.1 (0.8, 11.0) 0.089 | 0.713 |
| Yes | 1.9 (0.9, 9.0) 0.090 |  |
| AJCC. stage |  | 0.012 |
| I | 1.4 (0.7, 2.9) 0.128 |  |
| II | 2.9 (1.3, 5.2) 0.022 |  |
| III | **2.4 (1.2, 3.9) <0.001** |  |
| IV | **5.8 (2.1, 14.3) <0.001** |  |
| TP53 |  | 0.122 |
| Wild-type | **1.4 (1.1, 3.5) 0.022** |  |
| Mutation | **1.2 (1.0, 1.8) 0.026** |  |
| TERT |  | 0.751 |
| Wild-type | 1.9 (0.1, 3.5) 0.761 |  |
| Mutation | 0.9 (0.4, 1.9) 0.804 |  |
| ARID2 |  | 0.512 |
| Wild-type | 1.3 (0.9, 3.1) 0.123 |  |
| Mutation | 0.8 (0.4, 1.6) 0.477 |  |
| ARID1A |  | 0.042 |
| Wild-type | **1.2 (1.0, 4.2) 0.043** |  |
| Mutation | **1.9 (1.2, 3.2) 0.011** |  |

Significant P values are in bold;
