# Supplementary material for: Genetic Alterations and Transcriptional Expression of m6A RNA Methylation Regulators Drive a Malignant Phenotype and Have Clinical Prognostic Impact in Hepatocellular Carcinoma
Source: Front Oncol. 2020 Jul 21;10:900. doi: 10.3389/fonc.2020.00900 (PMC7396691; doi:10.3389/fonc.2020.00900)
Supplement: Table S7 — Univariate analysis for overall survival of patients stratified by the status of m6A regulatory gene alterations in addition to (A) TP53, (B) TERT, and (C) ARID1A mutation status. [file Table_7.DOCX]

**Table S7.** Univariate analysis for overall survival of patients stratified by the status of m6A regulatory gene alterations in addition to (A) TP53, (B) TERT, and (C) ARID1A mutation status.

| Parameters and model | Overall survival | |
| --- | --- | --- |
|  | Hazard ratio (95% CI) | P value |
| TP53 WT & unaltered m6A regulatory genes | ref | ref |
| TP53 WT & altered m6A regulatory genes | 1.3 (1.0, 4.2) | 0.033 |
| TP53 mutant & altered m6A regulatory gene(s) | 2.7 (1.1, 5.2) | 0.001 |
| TERT WT & unaltered m6A regulatory genes | ref | ref |
| TERT mutant & unaltered m6A regulatory genes | 1.3 (1.0, 3.1) | 0.042 |
| TERT WT & altered m6A regulatory gene(s) | 2.3 (1.1, 3.4) | 0.001 |
| ARID1A WT & unaltered m6A regulatory genes | ref | ref |
| ARID1A mutant & unaltered m6A regulatory genes | 1.2 (1.1, 3.4) | 0.006 |
| ARID1A WT & altered m6A regulatory gene(s) | 4.5 (1.2, 9.1) | 0.009 |

WT: wild-type;
