# Supplementary material for: Genetic Alterations and Transcriptional Expression of m6A RNA Methylation Regulators Drive a Malignant Phenotype and Have Clinical Prognostic Impact in Hepatocellular Carcinoma
Source: Front Oncol. 2020 Jul 21;10:900. doi: 10.3389/fonc.2020.00900 (PMC7396691; doi:10.3389/fonc.2020.00900)
Supplement: Table S8 — Multivariate analysis for overall survival in HCC patients. [file Table_8.DOCX]

**Table S8.** Multivariate analysis for overall survival in HCC patients.

|  | Model I | | Model II* | |
| --- | --- | --- | --- | --- |
|  | HR (95%CI) | P value | HR (95%CI) | P value |
| AJCC. stage |  |  |  |  |
| I | ref | ref | ref | ref |
| II | 1.2 (0.8, 2.3) | 0.209 | 1.5 (0.8, 2.9) | 0.243 |
| III | **2.3 (1.5, 3.9)** | **<0.001** | **2.3 (1.2, 3.7)** | **<0.001** |
| IV | **5.7 (2.2, 11.1)** | **<0.001** | **5.5 (2.2, 13.1)** | **<0.001** |
| TP53 |  |  |  |  |
| Wild-type | ref | ref | - | - |
| Mutation | **1.2 (1.0, 1.8)** | **0.026** | **-** | **-** |
| ARID1A |  |  |  |  |
| Wild-type | ref | ref | ref | ref |
| Mutation | **1.8 (1.1, 4.2)** | **0.020** | **1.7 (1.1, 4.3)** | **0.026** |
| M6A regulatory genes | ref | ref | ref | ref |
| Altered | ref | ref |  |  |
| Unaltered | 1.7 (0.8, 3.4) | 0.103 | **1.8 (1.2, 4.4)** | **0.010** |

Model I included TP53 mutation; Model II* excluded TP53 mutation; HR: hazard ratio
